# Association of selected SNP with carcass and taste panel assessed meat quality traits in a commercial population of Aberdeen Angus-sired beef cattle

**DOI:** 10.1186/1297-9686-41-36

**Published:** 2009-06-25

**Authors:** Jennifer L Gill, Stephen C Bishop, Caroline McCorquodale, John L Williams, Pamela Wiener

**Affiliations:** 1 The Roslin Institute and Royal (Dick) School of Veterinary Studies, The University of Edinburgh, Roslin BioCentre, Roslin, Midlothian EH25 9PS, UK; 2 Parco Tecnologico Padano, Via Einstein, Polo Universitario, Lodi 26900, Italy

## Abstract

**Background:**

The purpose of this study was to evaluate the effects of eight single nucleotide polymorphisms (SNP), previously associated with meat and milk quality traits in cattle, in a population of 443 commercial Aberdeen Angus-cross beef cattle. The eight SNP, which were located within five genes: μ-calpain (*CAPN1*), calpastatin (*CAST*), leptin (*LEP*), growth hormone receptor (*GHR*) and acylCoA:diacylglycerol acyltransferase 1 (*DGAT1*), are included in various commercial tests for tenderness, fatness, carcass composition and milk yield/quality.

**Methods:**

A total of 27 traits were examined, 19 relating to carcass quality, such as carcass weight and fatness, one mechanical measure of tenderness, and the remaining seven were sensory traits, such as flavour and tenderness, assessed by a taste panel.

**Results:**

An SNP in the *CAPN1 *gene, *CAPN316*, was significantly associated with tenderness measured by both the tenderometer and the taste panel as well as the weight of the hindquarter, where animals inheriting the CC genotype had more tender meat and heavier hindquarters. An SNP in the leptin gene, *UASMS2*, significantly affected overall liking, where animals with the TT genotype were assigned higher scores by the panellists. The SNP in the *GHR *gene was significantly associated with odour, where animals inheriting the AA genotype produced steaks with an intense odour when compared with the other genotypes. Finally, the SNP in the *DGAT1 *gene was associated with sirloin weight after maturation and fat depth surrounding the sirloin, with animals inheriting the AA genotype having heavier sirloins and more fat.

**Conclusion:**

The results of this study confirm some previously documented associations. Furthermore, novel associations have been identified which, following validation in other populations, could be incorporated into breeding programmes to improve meat quality.

## Background

Meat quality is of great importance to the beef industry where the consumer is willing to pay more for superior products [1]. Traditional trait improvement has centred on quantitative genetics, using statistical analysis of phenotypic data to determine animals with the highest genetic merit [2]. This selection approach is most effectively implemented for highly heritable traits that are easily recorded before reproductive age. However, meat quality traits can usually only be measured post-slaughter and often have low heritabilities [3], therefore making progress using direct measurement is difficult for these traits. Marker-assisted selection has the potential to significantly increase the rate of genetic improvement in such traits [4], using markers linked to economically relevant traits, which can be used to predict the genetic merit of an animal. Several such markers have been identified in the last decade. These include markers based on polymorphisms in the leptin (*LEP*) gene, involved in the control of appetite and energy metabolism, which have been shown to be associated with carcass fat [5-8], body weight [6], and growth rate [9]; polymorphisms in the μ-calpain (*CAPN1*) and calpastatin (*CAST*) genes, which are known to play a key role in post mortem tenderisation of meat and have been associated with meat tenderness [10-13]; and polymorphisms in the bovine growth hormone receptor (*GHR*) gene, which have been associated with drip loss [14], body weight [15,16] and marbling score [17]. Before such marker information can be used in breeding programmes, it is important that unbiased and independent validation studies in different breeds are carried out to establish whether observable effects are found in the breed/population under selection.

The aim of this study was to test such associations between eight single nucleotide polymorphisms (SNP) from five different genes and meat quality traits in a sample of Aberdeen Angus-cross animals collected in Scotland. All the tested SNP have been previously reported to be associated with various carcass and meat quality traits in cattle or pigs and have been incorporated into commercially available tests for meat or milk quality.

## Methods

### Sample collection

Commercial crossbred beef cattle (n = 443 animals) with purebred Aberdeen Angus sires were sourced through the Scotbeef abattoir (Bridge of Allan, Scotland). Cattle originated from 14 breeder finisher farms (*i.e*. farms where animals are bred and finished on the same farm) and were selected to be representative of British commercial cattle slaughtered for beef production, being a mix of heifers and steers ranging between 408 and 912 days old at kill, with the age differences depending largely on the farm. The 443 animals used in the experiment included 135 females and 308 males. The sires for all animals were pure-bred Aberdeen Angus whilst the dams were a mixture of purebreds of various breeds and crossbreeds including Aberdeen Angus, Aberdeen Angus-cross, Simmental-cross and Limousin-cross.

Cattle were stunned by captive bolt before being slaughtered by exsanguination and dressed using standard commercial specifications. During exsanguination, 100 mL blood was collected and frozen for DNA extraction.

### Carcass trait measurement

At slaughter, hot carcass weight was recorded and carcasses were graded by a Meat Hygiene Service assessor for muscle composition and carcass fatness according to the standard European Union beef carcass classification scale (EUROP) [18]. Conformation and fat class scores were transformed into a 7-point numerical scale [19]. Twenty-four hours after slaughter, pH and temperature were recorded in the sirloin muscle with the TESTO 205 pH meter (TESTO, Hampshire, UK) and ETI FPT thermometer (ETI Ltd. Worthing, UK), respectively.

At deboning, weight of the hindquarter and sirloins were recorded. Sirloins were vacuum-packed and stored below 4°C for 21–30 d to mature, then removed from the vacuum pack, patted dry to remove excess moisture and weighed. Three steaks were cut from the centre of the sirloin as follows: for tenderometer testing 3–4 cm thick, for sirloin measurements 1–2 cm thick and for sensory testing 2 cm thick.

For tenderometer testing, steaks were trimmed to 200–220 g of eye muscle and placed in a water bath at 100°C until the centre of the sample reached 82°C. Samples were left to cool to 7°C then tested using a MIRINZ Tenderometer machine (AgResearch, Hamilton, New Zealand).

A full list of analysed carcass quality traits can be found in Table 1.

**Table 1:** Carcass trait means, coefficients of variation and heritability estimates (adapted from: Gill, Matika, Williams, Worton, Wiener, and Bishop: Consistency statistics and genetic parameters for taste panel assessed meat quality traits in a commercial population of Angus-sired beef cattle (submitted))

Trait	Mean	CV	h^2 ^(se)^2^
Tenderometer score, kPa^1^	24.69	20.33	0.30 (0.22)
Hot carcass weight, kg	319.7	12.0	0.70 (0.28)
Sirloin weight after maturation, kg	7.10	14.0	0.24 (0.18)
Conformation class, transformed numerical scale	7.11	21.43	0.32 (0.20)
Eye muscle length as a % of sirloin length	77.45	10.73	0.09 (0.15)
Eye muscle area, mm^2^	10870	15.0	-
Eye muscle depth, mm	69.51	12.01	-
Eye muscle length, mm	156.3	8.38	0.03 (0.12)
Fat class, transformed numerical scale	8.61	11.74	0.11 (0.14)
Sirloin fat depth, mm	6.41	51.54	0.30 (0.20)
Gristle encroachment, mm	20.73	41.08	0.14 (0.18)
Gristle distance from eye muscle base, mm	53.55	20.01	0.21 (0.21)
Gristle distance from fat band, mm	14.23	43.87	0.13 (0.18)
Gristle length, mm	69.23	20.75	-
Sirloin weight as % of hindquarter weight	9.71	10.18	0.45 (0.23)
Sirloin steak tail length, mm	46.95	42.71	0.13 (0.17)
Temperature at 24 h, °C	4.06	14.25	-
Hindquarter weight, kg	73.44	11.04	0.23 (0.20)
Sirloin weight before maturation, kg	7.14	14.21	0.24 (0.18)
pH at 24 h	5.56	2.40	0.02 (0.14)

^1 ^Units for tenderometer score are kilopascals

^2 ^Where heritability values are absent the variance converged at a boundary

### Taste panel selection and assessments

Taste panel members were chosen among workers at the Scotbeef meat processing plant in East Kilbride, Scotland. Members of staff (n = 38) were tested using the Triangle and Matching tests [20] with 10 being discarded due to poor scores. Taste panels included six members and an average of nine samples were tested in one sitting with the addition of one blind repeat steak per panel. Participants were instructed to rinse their mouths with water before tasting began as well as between samples. They were also instructed not to eat or drink for one hour prior to the test.

Prior to assessment sirloin steaks were cooked using a Lincat Lynx 400 electric griddle (Lincat Ltd, Lincoln, UK) until a thermometer placed in the centre of the steak reached 74°C. The six panellists then scored the steaks on a 1–8 scale for seven sensory traits, a full list of which can be seen in Table 2 along with an explanation of the scoring scheme used. In total there were 49 taste panel sittings. Taste panel members participated in one to 37 panels with an average of eight sittings per panellist.

**Table 2:** Means, coefficients of variation and heritability estimates for taste panel assessed sensory traits (adapted from: Gill, Matika, Williams, Worton, Wiener, and Bishop: Consistency statistics and genetic parameters for taste panel assessed meat quality traits in a commercial population of Angus-sired beef cattle (submitted))

Trait	1	8	Mean	CV	h^2 ^(se)^1^
Abnormal flavour	Extremely strong	Extremely weak	6.24	12.34	-
Abnormal odour	Extremely strong	Extremely weak	6.36	11.32	-
Flavour	Extremely weak	Extremely strong	5.54	9.67	-
Odour	Extremely weak	Extremely strong	5.26	10.25	-
Juiciness	Extremely dry	Extremely juicy	5.52	12.33	0.15 (0.11)
Tenderness	Extremely tough	Extremely tender	5.72	11.37	0.06 (0.13)
Overall liking	Disliked extremely	Liked extremely	5.62	10.97	0.16 (0.16)

^1 ^Where heritability values are absent the variance converged at a boundary

### Paternity determination

Due to the possibility of multiple sire mating and discrepancies between the recorded and the true sire, paternity was determined using genetic markers. Details are described in full in: Gill, Matika, Williams, Worton, Wiener, and Bishop: Consistency statistics and genetic parameters for taste panel assessed meat quality traits in a commercial population of Angus-sired beef cattle (submitted).

Briefly, genotypes were obtained for each sample for a panel of 15 unlinked microsatellite markers. Genotypes for each offspring and all possible sires were analyzed with the program Cervus [21], which assigns paternity using a likelihood method. There were 69 offspring whose sires could not be determined, therefore the sire was set to "unknown" in the pedigree, however, the phenotypes of these samples were retained in the analyses.

### SNP genotyping

Samples were genotyped at eight SNP from five different genes by Orchid Cellmark Ltd (Oxfordshire, UK).

The SNP locations in each gene, Genbank accession number and the positions (intron/exon etc) are listed in Table 3. In brief the genes were *CAPN1 *(2 SNP), *CAST *(1 SNP), leptin (3 SNP), *GHR *(1 SNP), and acylCoA:diacylglycerol acyltransferase 1 (*DGAT1*) (1 dinucleotide substitution). All animals with phenotypes were genotyped, as were all available sires.

**Table 3:** SNP name and location

Gene	BTA	SNP name	Location	GenBank Accession number and base position	SNP
*CAPN1*^1^	29	*CAPN316*	Exon 9	AF252504-5709	G/C
		*CAPN 4751*	Intron 17 between exon 17 and 18	AF248054-6545	C/T
*CAST*^2^	7	*UoGCAST*	Intron 5 between exon 5 and 6	AY008267-282	G/C
*LEP*^3^	4	*UASMS1*	Leptin promoter region	AB070368-207	C/T
		*UASMS2*	Leptin promoter region	AB070368-528	C/T
		*Exon2FB*	Exon 2	AY138588-305	C/T
*GHR*^4^	20	*F279Y*	Exon 8	AY748827-914	A/T
*DGAT1*^5^	14	*K232A*	Exon 8	AY065621-6829 AY065621-6830	A/G A/C

^1 ^Calpain was mapped by Smith *et al*., (2000) [32], *CAPN316 *SNP reported by Page *et al.*, (2002) [35] and *CAPN4751 *SNP reported by White *et al.*, (2005) [13]

^2 ^Calpastatin was mapped by Bishop *et al.*, (1993) [36] and the SNP was reported by Schenkel *et al*., (2006) [11]

^3 ^Leptin was mapped by Stone *et al.*, (1996) [37], *UASMS1 *and *UASMS2 *SNP reported by Nkrumah *et al.*, (2005) [9], *Exon2FB *SNP reported by Buchanan *et al.*, (2002) [5]

^4 ^Growth hormone receptor was mapped by Moody *et al.*, (1995) [43], SNP reported by Blott *et al.*, (2003) [42]

^5 ^acylCoA:diacylglycerol acyltransferase 1 was mapped by Cases *et al.*, (1998) [39], dinucleotide substitution reported by Grisart *et al.*, (2002) [40]

#### Multiplex PCR of loci for SNP genotyping

A single PCR was used to generate 12 amplicons. Each 10 μL reaction volume contained 5 μL DNA, 100 μM of each dNTP, 1 × QIAGEN PCR buffer which contained 1.5 mM MgCl_2_, 1× primer mix, 1 U Hot Start Taq (QIAGEN, UK) and 0.1 μg/μL BSA. PCR conditions consisted of 15 min at 95°C followed by 32 cycles of 30 s as 94°C, 1 min at 67°C and 1 min at 72°C. Following amplification, 4 μL of a solution containing 4 U Exonuclease I (NEB, UK), 1.4 μL of 10× Antarctic Phosphatase buffer (NEB, UK) and 2 U Antarctic Phosphatase (NEB, UK) was added to the PCR product. Samples were then incubated for 60 min at 37°C followed by 15 min at 72°C.

#### Single base extension

SNaPshot^® ^(Applied Biosystems, UK) reactions included 5 μL of Antarctic Phosphatase/Exonuclease I treated PCR product, 2 μL of SNaPshot Multiplex Ready Reaction Mix (Applied Biosystems, UK), 2 μL H_2_O and 1 μL probe mix. Thermocycling conditions consisted of 30 s at 94°C and 20 s at 67°C. Following single base extension, 4 μL of CIP solution containing 0.4 μL of 10× NEBuffer 3 (NEB, UK), and 2 U CIP (NEB, UK) was added to each sample. The samples were then incubated at 37°C for 60 min followed by 85°C for 20 min.

#### Electrophoresis and scoring

Five μL of the SNaPshot/CIP product was added to 10 μL of Hi-Di™ formamide (Applied Biosystems, UK) and the samples were electrophoresed on a 3100 Genetic Analyzer (Applied Biosystems, UK). Genemapper^® ^v4.0 was used to interpret the genetic profiles.

### Data analysis

#### SNP frequencies and linkage disequilibrium

Genotype frequencies of each polymorphism were tested for deviations from Hardy-Weinberg equilibrium by *χ*^2 ^tests [22] (significance based on *P *< 0.05). Pairwise genotype combinations of the SNP were also tested for linkage disequilibrium (LD), the degree of non-random association of alleles at two or more loci, using the Haploview program, version 4 [23]. The Haploview program uses a two-marker EM to estimate the maximum-likelihood values of the four gamete frequencies as well as D' and *r *^2 ^values and LOD scores.

#### Mixed model association analysis

The relationship between the different genotypes of each SNP and the various traits recorded was evaluated using a single-marker mixed-model association analysis. Data were analyzed by fitting a linear mixed model using the restricted maximum likelihood method (REML) provided in Genstat, release 10 [24]. The statistical model included fixed effects of farm, genotype, sex and the genotype-sex interaction, and random effects of sire, slaughter date (panel date for the taste panel traits), interaction of sire and slaughter date (panel date for the taste panel traits) and interactions of sire and slaughter date (panel date for the taste panel traits) with the genotype/sex groups. These latter interactions took into account the possibility of genotype/sex effects varying with sire or slaughter date (panel date for the taste panel traits) or both. An additional term including animal ID and steak ID (A or B) was added for analysis of taste panel traits to allow a distinction to be made between the A and B steaks of those animals that had repeat steaks tested.

The general model used for carcass traits was as follows:



where:

Y_*ijklmn *_is the trait measured on the individual i

μ is the overall mean for the trait

F_*j *_is the fixed effect of farm *j *(14 levels)

G_*k *_is the fixed effect of SNP genotype *k *(3 levels)

S_*l *_is the fixed effect of sex *l *(2 levels)

(G × S)_*kl *_is the interaction between the k-th SNP genotype and the l-th sex

K_*m *_is the random effect of kill-date *m*

M_*jn *_is the random effect of the n-th sire on the j-th farm

e_*jklmno *_is the residual term associated with the observation

Additional interaction terms between sire, kill-date, genotype and sex groupings were fitted as random effects. Variance components were constrained to be non-negative, *i.e*. where effects were estimated to be negative they were set to zero.

For the taste panel traits the general model was as follows:



where additional terms are:

P_*m *_is the random effect of taste panel date *m*

T_*io *_is the random effect of the o-th steak (A or B) for the i-th animal

e_*ijklmnop *_is the residual error associated with the observation.

Again, additional interaction terms between sire, taste panel date, genotype and sex groupings were fitted as random effects, and variance components were constrained to be non-negative.

The effects of several covariates (percentage Aberdeen Angus, hot carcass weight and age at kill) were also examined in separate analyses. The percentage Aberdeen Angus (% AA) was based on dam breed so that each animal was assigned a value of 100% (if the dam was AA), 75% (if the dam was AA-cross) or 50% (if the dam was neither). Statistical significance for the fixed effects was determined using approximate F-statistics with denominator degrees of freedom [25] estimated in the Genstat REML procedure.

Additive effects and dominance deviation were also calculated using a re-parameterized model. The additive effect was estimated as the difference between the mean of the two homozygotes divided by two, and dominance was estimated as the deviation of the heterozygote from the mean of the two homozygotes [22].

#### Correction for multiple testing

To allow correction for the fact that a large number of traits were analysed with a large number of SNP, and hence a high probability of false positive results, a Bonferroni correction was applied. The three leptin SNP and the two μ-calpain SNP were found to be in partial or strong LD so that the effective number of SNP tested was estimated as five. The correction for multiple SNP testing resulted in an adjusted *P *value of 0.01 for the 5% significance level.

#### Haplotype reconstruction and analysis

Haplotypes were reconstructed for the genes that contained more than one SNP using software that determines the gametic haplotypes for each animal where phase is known with certainty [26] based on sire and sibling genotype information. Haplotype pairs were unambiguously reconstructed for 258 individuals for SNP in the leptin gene and 291 individuals for SNP in the *CAPN1 *gene out of the 443 genotyped animals. In order to determine whether the haplotype information accounted for additional variation beyond the SNP genotype analysis, we nested the haplotype group (a combination of the two haplotypes) within a SNP model *i.e*. the model was the same as the genotype model but with additional fixed terms accounting for the variation between the haplotype groups within the SNP groups in the fixed model. This analysis was carried out for each of the traits found to be significantly affected by either the μ-calpain or leptin SNP, *i.e*. tenderometer, weight of hindquarter and tenderness for the μ-calpain markers and overall liking for the leptin markers. Statistical significance of the extra variation accounted for by the presence of the haplotype groups in the model was determined using approximate F-statistics derived from Wald statistics with denominator degrees of freedom estimated in the Genstat REML procedure [25].

## Results

### Genotype and allele frequencies

A total of 443 animals were genotyped at all eight SNP, however, successful genotype assignment was not possible for all animals, in particular for the leptin SNP *UASMS1 *where 17 animals were missing genotypes (Table 4). The frequencies of genotypes at all eight SNP were in agreement with Hardy-Weinberg equilibrium [22]. The two SNP in the *CAPN1 *gene and the three SNP in the leptin gene were found to be in LD with D' above 0.62 for each SNP pair combination. R^2 ^values between each pair of leptin SNP were above 0.18 whilst the value between the two *CAPN1 *SNP was 0.07.

**Table 4:** Genotypic and allelic frequencies for SNP

SNP	Genotype	Genotype frequency	Allele	Allele frequency
*CAPN4751*	CC (n = 182)	0.41	C	0.65
	CT (n = 209)	0.48	T	0.35
	TT (n = 48)	0.11	-	-
*CAPN316*	CC (n = 20)	0.05	C	0.22
	CG (n = 152)	0.35	G	0.78
	GG (n = 268)	0.61	-	-
*Exon2FB*	CC (n = 130)	0.29	C	0.55
	CT (n = 222)	0.50	T	0.45
	TT (n = 90)	0.20	-	-
*UASMS2*	CC (n = 166)	0.38	C	0.63
	CT (n = 219)	0.50	T	0.37
	TT (n = 54)	0.12	-	-
*UASMS1*	CC (n = 77)	0.18	C	0.42
	CT (n = 205)	0.48	T	0.58
	TT (n = 144)	0.34	-	-
*DGAT1*	AA (n = 22)	0.05	A	0.25
	AG (n = 176)	0.40	G	0.75
	GG (n = 243)	0.55	-	-
*GHR*	AA (n = 12)	0.03	A	0.13
	AT (n = 93)	0.21	T	0.87
	TT (n = 333)	0.76	-	-
*UoGCAST1*	CC (n = 181)	0.41	C	0.64
	CG (n = 207)	0.47	G	0.36
	GG (n = 54)	0.12	-	-

### Phenotypic trait data

An analysis of the phenotypic trait data is presented in full in: Gill, Matika, Williams, Worton, Wiener, and Bishop: Consistency statistics and genetic parameters for taste panel assessed meat quality traits in a commercial population of Angus-sired beef cattle (submitted), including consideration of the reliability of taste panel measurements and genetic parameters for traits of interest. In brief, a total of 27 traits were measured, seven of which are taste panel assessed sensory traits, one a mechanical measure of tenderness with the remaining 19 being carcass and sirloin measurements. Trait means, coefficients of variation and heritability estimates are given in Tables 1 and 2.

### Genotype effects

Genotypes for four of the eight SNP tested did not significantly influence any of the 27 traits measured and 20 of the traits were not affected by the genotypes of any of the SNP tested. Tenderometer score, sirloin weight after maturation, sirloin fat depth, hindquarter weight, odour, overall liking and tenderness showed significant additive effects of at least one of the remaining four SNP.

At the *CAPN1 *gene SNP, *CAPN316*, the C allele was associated with reduced tenderometer values, increased hindquarter weight and an increase in taste panel assessed tenderness compared to the G allele (Table 5). The estimated differences between the homozygous genotypes CC and GG were 2.93 kPa, 3.83 kg and 0.37 taste panel units for shear force, hindquarter weight and taste panel tenderness respectively. There were no significant dominance effects seen for *CAPN316*.

**Table 5:** Genotype means, standard errors, P values and estimates of additive and dominance effects for SNP with significant trait associations

	SNP	Genotype means ± se^1^			Overall P-value^2^	a ± se^1,3^	d ± se^1,4^
	*CAPN316*	CC	CG	GG			

Tenderometer score, kPa^5^		22.25 ± 1.16	24.24 ± 0.7	25.18 ± 0.67	0.01^6^	1.46 ± 0.52**	0.53 ± 0.61
Hindquarter weight, kg		75.67 ± 1.57	72.05 ± 0.84	71.84 ± 0.77	0.04	-1.92 ± 0.76*	-1.70 ± 0.92
Tenderness		6.00 ± 0.16	5.79 ± 0.08	5.63 ± 0.07	0.01^6^	-0.19 ± 0.08*	-0.03 ± 0.09

	*UASMS2*	CC	CT	TT			

Overall liking		5.59 ± 0.08	5.55 ± 0.08	5.80 ± 0.10	0.02	0.10 ± 0.05*	-0.15 ± 0.06*

	*DGAT1*	AA	AG	GG			

Sirloin weight after maturation, kg		8.31 ± 0.46	7.17 ± 0.12	7.14 ± 0.11	0.04	-0.59 ± 0.23*	-0.55 ± 0.23*
Sirloin fat depth, mm		11.11 ± 1.65	6.62 ± 0.4	6.53 ± 0.33	0.05	-2.29 ± 0.83*	-2.20 ± 0.87 *

	*GHR*	AA	AT	TT			

Odour		5.64 ± 0.19	5.24 ± 0.08	5.16 ± 0.06	0.02	-0.24 ± 0.09**	-0.16 ± 0.11

^1 ^Mean values were adjusted for farm and averaged over sex. Estimate of the effect is expressed in units of the trait

^2 ^*P*-value for the overall genotype effect

^3 ^Additive effect is estimated as the difference between the 2 homozygous means divided by 2

^4 ^Dominance effect is estimated as the non-additive genetic effects or the deviation of the heterozygote from the mean of the 2 homozygotes

** *P *< 0.01 and * *P *< 0.05

^5 ^Units for tenderometer score are kilopascals

^6 ^Associations remain significant following correction for multiple testing (adjusted P-value was 0.01)

The T allele of the leptin SNP, *UASMS*2, was significantly associated with an increase in overall liking, with the estimated difference between the homozygous genotypes TT and CC equal to 0.21 taste panel units (Table 5). There was a significant dominance effect (*P *= 0.01) such that the C allele was dominant to the T allele.

For *DGAT1*, the A allele was associated with an increase in sirloin weight after maturation and an increase in sirloin fat depth when compared to the G allele (Table 5). The estimated differences between the homozygous genotypes AA and GG were 1.17 kg and 4.58 mm for sirloin weight after maturation and sirloin fat depth. There were significant dominance effects for both sirloin weight after maturation and sirloin fat depth (*P *= 0.02 and 0.01, respectively). Animals with the heterozygous genotype had values close to the homozygous GG animals, indicating a strong degree of dominance of the G allele over the A allele.

The A allele of the *GHR *SNP was significantly associated with an increase in taste panel-assessed odour, with the estimated difference between the homozygous genotypes AA and TT equal to 0.48 taste panel units (Table 5). There was no significant dominance effect.

To further investigate the observed associations, a number of covariates were added to the fixed model. These included hot carcass weight, age at kill and percentage Aberdeen Angus (50, 75 or 100%, depending on dam breed). Percentage Aberdeen Angus made no difference to the genotypic effects for all of significant traits. As expected, when hot carcass weight was fitted as a covariate, the significance of the associations with weight traits was reduced. Age at kill made no difference to the effects of genotype on tenderness, hindquarter weight, overall liking, sirloin fat depth or odour. The only SNP trait association affected by age at kill was the association between *DGAT1 *genotype and sirloin weight after maturation. The p-value increased above 0.05 although animals with the AA genotype were still found to have heavier sirloins after maturation than those animals with either AT or TT genotypes. Most age differences between animals are accounted for in the model as farm effects, hence the small impact of this covariate.

#### Sex × genotype interactions

There were significant genotype-by-sex interactions for six of the significant trait SNP genotype pairs (Table 6). These were the *CAPN316 *association with tenderometer, weight of hindquarter and taste panel tenderness; the *GHR *association with odour; and the *DGAT1 *association with complete sirloin weight and sirloin fat depth. When the genotype means of each sex were assessed using pair wise comparisons it became apparent that the majority of significant genotype-trait associations mentioned previously were stronger in the female animals. Females with the CC genotype at the *CAPN316 *SNP had significantly lower tenderometer values, higher hindquarter weights and higher taste panel tenderness scores than CG or GG animals. In contrast, the CC males had higher tenderometer values than the other genotypes although this difference was not significant.

**Table 6:** Genotype means and standard errors for each genotype by sex group

			Genotype means ± se^1^					
			
	Sex	Genotype	Tenderometer score, kPa^2^	Weight of hindquarter, kg	Tenderness	Odour	Sirloin weight after maturation, kg	Sirloin fat depth, mm
*CAPN316*	F	CC	19.51 ± 1.60^a^	73.17 ± 2.24^a^	6.43 ± 0.23^a^	---	---	---
		CG	25.14 ± 0.84^b^	66.04 ± 1.12^b^	5.75 ± 0.1^b^	---	---	---
		GG	25.74 ± 0.77^b^	67.27 ± 1.0^b^	5.58 ± 0.09^b^	---	---	---
	M	CC	24.99 ± 1.38^a, b^	78.17 ± 1.91^a^	5.57 ± 0.19^a^	---	---	---
		CG	23.34 ± 0.78^a^	78.06 ± 0.95^a^	5.82 ± 0.09^a^	---	---	---
		GG	24.60 ± 0.73^b^	76.4 ± 0.86^a^	5.68 ± 0.08^a^	---	---	---
*GHR*	F	AA	---	---	---	6.13 ± 0.34^a^	---	---
		AT	---	---	---	5.34 ± 0.11^b^	---	---
		TT	---	---	---	5.11 ± 0.07^b^	---	---
	M	AA	---	---	---	5.15 ± 0.16^a^	---	---
		AT	---	---	---	5.14 ± 0.08^a^	---	---
		TT	---	---	---	5.21 ± 0.07^a^	---	---
*DGAT1*	F	AA	---	---	---	---	9.02 ± 0.88^a^	15.7 ± 3.22^a^
		AG	---	---	---	---	6.85 ± 0.16^b^	6.66 ± 0.53^b^
		GG	---	---	---	---	6.73 ± 0.13^b^	6.46 ± 0.43^b^
	M	AA	---	---	---	---	7.59 ± 0.22^a^	6.56 ± 0.8^a^
		AG	---	---	---	---	7.5 ± 0.13^a^	6.58 ± 0.46^a^
		GG	---	---	---	---	7.54 ± 0.13^a^	6.60 ± 0.42^a^

^1 ^Mean values were adjusted for farm. Estimate of the effect is expressed in units of the trait

^a, b, ^Within a trait and sex, genotype means without a common superscript letter are statistically significantly different (P < 0.05)

^2 ^Units for tenderometer score are kilopascals

Additionally there was a significant difference in odour score assigned to female animals with the AA, AT and TT genotypes at the *GHR *SNP. Here, AA animals had significantly higher scores than either AT or TT animals. For the male animals, those with the TT genotype received the highest score for odour, although again, the differences between genotypes were not significant. Finally, female animals with the AA genotype at the *DGAT1 *SNP had significantly heavier sirloins after maturation and sirloin fat depths than AG or GG animals. For males the AA animals also had heavier sirloins although the GG animals had higher values of fat depth, however, differences between genotypes were not significant.

### Haplotype analysis

Haplotypes with a frequency of ≤ 0.01 were excluded from the analysis; this left four haplotypes for SNP in the *CAPN1 *gene (CC, CG, TC and TG (*CAPN4751*, *CAPN316*)), and four haplotype possibilities for SNP in the leptin gene (CCT, CTT, TCC and TTT (*Exon2FB, UASMS2, UASMS1*)) (Table 7). Results from the nested haplotype model for each of the significant μ-calpain and leptin traits are shown in Table 8. In all cases F-statistics were not significant and, in fact, close to unity, showing that haplotype information does not account for additional variation compared to a model with only genotype information.

**Table 7:** Haplotype frequencies for calpain and leptin

Gene	Haplotype	Frequency
Calpain^1^	CC	0.16
	CG	0.52
	TC	0.02
	TG	0.30
Leptin^2^	CCT	0.19
	CTT	0.35
	TCC	0.44
	TTT	0.02

^1 ^Calpain haplotype: *CAPN4751*, *CAPN316*

^2 ^Leptin haplotype: *Exon2FB*, *UASMS2*, *UASMS1*

**Table 8:** F-statistics and degrees of freedom for SNP-by-haplotype interaction term for a nested model of genotype effects

Gene	SNP	Trait	F-Statistic (d.f.: numerator, denominator)
Calpain	*CAPN316*	Tenderometer score, kPa^1^	1.16 (8, 79.8)^ns^
		Weight of hindquarter, kg	0.88 (8, 32.4)^ns^
		Tenderness	0.85 (8, 120.4)^ns^
Leptin	*UASMS2*	Overall Liking	0.95 (11, 197.5)^ns^

** *P *< 0.01, * *P *< 0.05 and^ ns ^*P *> 0.05

^1^Units for tenderometer score are kilopascals

## Discussion

The primary objective of this study was to test previously identified associations between SNP from five genes and economically important meat quality traits. The SNP tested are located in the *CAPN1*, *CAST*, *DGAT1*, leptin and *GHR *genes and have been incorporated into commercially available genetic tests based on previously reported associations with meat quality or carcass traits. The two *CAPN1 *SNP, (*CAPN316 *and *CAPN4751*) and the *CAST *SNP make up the Igenity *Tender*GENE panel [27], with the two *CAPN1 *SNP forming the basis of the GeneSTAR tenderness test [28]. Additionally, SNP in the leptin gene are included in the Igenity *Opti*GRID test, whilst the SNP in the *DGAT1 *and *GHR *genes comprise the Igenity *Opti*YIELD test [27].

### Allele frequencies

The allele frequencies found for the *CAPN316*, *CAPN4751 *and *UoGCAST *SNP are in agreement with previous studies [7,29-31]. Allele frequencies were also estimated for the sire population (purebred Aberdeen Angus). On the whole, frequencies in the sire population were found to be similar to those in the progeny, however, at the *CAPN316 *SNP, the C allele was found to have a frequency of only 0.10 in the sires (data not shown), as opposed to 0.22 in the offspring. This is due to the absence of sires with the CC genotype at this SNP.

### Single genotype-trait associations

Using single-marker, mixed-model association analysis four of the SNP were found to be associated with one or more of the traits tested in a significantly additive manner. These include the *CAPN316 *SNP in the *CAPN1 *gene, the *UASMS2 *SNP in the leptin gene, the *DGAT1 *SNP and the *GHR *SNP.

The *CAPN1 *gene, mapped to BTA 29 [32], encodes a cysteine protease thought to be the primary enzyme in the post mortem tenderisation of meat [33]. It is therefore a prime candidate for association studies involving tenderness, and, indeed, SNP in the gene have previously been associated with the trait in several studies [10,13,31,34,35]. In the present study the effects of two SNP located in *CAPN1 *were evaluated; *CAPN316*, situated in exon 9 of the gene, results in an amino acid substitution from alanine to glycine for the C and G alleles respectively; and *CAPN4751*, situated in the intron between the 17^th ^and 18^th ^exons of the gene. A significant effect of *CAPN316 *genotype was found on the hindquarter weight; animals with the CC genotype at this locus had significantly heavier hindquarters (by 3.8 kg) than GG animals (*P *= 0.04). However, some of this effect could be explained by effects on overall carcass weight. Additionally, animals inheriting two copies of the C allele at the *CAPN316 *SNP had meat that was more tender than animals with one or zero copies, when measured by both the tenderometer machine and the taste panel. The association with mechanical tenderness is consistent with work done in experimental herds of crossbred cattle [12,35].

Previous work has shown an association between genotypes at the other *CAPN1 *SNP, *CAPN4751*, and WBSF values in an experimental population and a *Bos indicus*-influenced crossbred population [13] where CC animals had significantly lower WBSF values, and therefore more tender meat, than TT animals. The current study did not find a significant effect of genotype at the *CAPN4751 *SNP on the tenderness trait (although the difference between CC and TT means was in the expected direction). Polymorphisms in *CAST*, an inhibitor of the *CAPN1 *protease mapped to BTA 7 [36], have previously been shown to be associated with tenderness [10,11,31]. However, the present study did not find a significant difference in tenderness, measured by the tenderometer machine, between the genotype groups.

Three SNP in the leptin gene, which has been mapped to BTA 4 [37] and produces a hormone that plays a key role in the regulation of appetite and body composition [38], were tested for associations in the present study. In contrast to previous studies [7,9], only one of these, *UASMS2*, was found to be significantly associated with any of the traits tested. Panellists gave animals with the TT genotype significantly higher overall liking scores than animals with CC or CT genotypes. A significant, non-additive association was also observed between the *UASMS2 *SNP and sirloin fat thickness, such that animals inheriting the CC genotype had significantly less fat surrounding the sirloin when compared to either CT or TT animals. This is in line with previous reports where *UASMS2 *was significantly associated with both backfat thickness and marbling score, with TT animals having higher values for both traits [9]. It may be that this association also explains the higher overall liking scores for TT animals as fat composition is known to affect meat flavour.

The *DGAT1 *gene, which has been mapped to BTA 14 [39], plays a key role in triglyceride synthesis [30]. The SNP studied here is an AA/GC dinucleotide substitution causing a *K *(lysine) to *A *(alanine) amino acid substitution (*K232A*) in the protein [40]. This polymorphism was shown to be significantly associated with milk fat yield and fat percentage where AA animals, with the lysine amino acid, had increased levels for both traits [40]. The present study found that the polymorphism was also associated with sirloin weight after maturation and sirloin fat depth. In both cases the A allele was associated with the higher value indicating that the increase in sirloin weight is probably due to the increase in the depth of fat surrounding the muscle. This is consistent with work done in German Holstein cattle where animals with the lysine allele at *DGAT1 *were found to have an increase in intramuscular fat content [30]. In contrast, work done on Brahman cattle found no association between *K232A *genotype and carcass fat traits [41].

The *GHR *polymorphism studied here is an A to T substitution in exon eight of the gene which results in a non-conservative replacement of phenylalanine with a tyrosine residue (*F*279*Y*) [42]. Whilst polymorphisms in the *GHR *gene (BTA 20 [43]) have previously been found to be significantly associated with drip loss [14], body weight [15,16] and USDA marbling score [17], the present study found no associations between *F279Y *genotype and any of the carcass quality traits. The only trait that was significantly affected by *GHR *genotype was meat odour as judged by the taste panel. The panellists assigned higher odour scores for animals with the AA genotype, which corresponds to the phenylanine amino acid, when compared to either AT or TT animals.

The present study confirmed some well-known associations and identified novel significant trait-genotype associations. However, there were some traits where associations were expected but not observed. Specifically, associations between carcass and sirloin related traits and the SNP in the leptin gene were expected but were not found. Serum levels of the leptin hormone have previously been found to be significantly associated traits such as marbling, backfat depth and kidney, pelvic and heart fat [44]. Additionally, genotype at the leptin SNP, *UASMS2*, has been shown to be associated with serum leptin concentration as well as with backfat thickness, marbling score and live weight at slaughter [9], carcass marbling score, Longissimus muscle (LM) area and hot carcass weight [45], although these results were not confirmed by Schenkel *et al*., (2005). As discussed above, the present study observed a significant, non-additive association between sirloin fat depth and *UASMS2 *genotype but no association was found with carcass or sirloin weight related traits. Previous reports also indicate associations between *UASMS1 *and fat yield and *Exon2FB *with fat and lean yield and grade fat [7], however, the current study found no associations between either *UASMS1 *or *Exon2FB *genotype and any of the traits tested. These contrasting results may be due to the different populations studied and indicate the importance of multiple validation studies in different breeds and populations.

### Sex × genotype interactions

The analyses of the mean trait value for each genotype in each sex for those trait-SNP pairs where there were significant sex interactions indicated that, for the majority of associations, the effect was primarily in the female animals. Differences between male and female genotype effects were seen in five traits: taste panel assessed tenderness, weight of hindquarter, odour, sirloin weight after maturation and sirloin fat depth. The reason for the difference between male and female animals is unknown. Whilst differences in meat quality between male and female cattle have been reported [46,47], there are few that describe differences in genetic effects between sexes such as those seen in the current study. Differences could be partly due to the limited number of females (135) in the analysis when compared to the males (308) although allele frequencies for both sexes were similar (data not shown). Alternatively, trait expression could be strongly correlated with fatness. Means for each sex showed that females tended to have higher fat class scores than males (data not shown). Therefore, it is possible the female animals are more likely to express genetic differences in traits that are correlated with fatness.

### Haplotype analysis

The haplotype analyses tested whether incorporating information on combinations of SNP (for μ-calpain and leptin respectively) led to an improvement over models with only single SNP genotypes. We found that using haplotypes in addition to SNP genotypes in the analysis accounted for no extra variation for any of the SNP/trait combinations. This may be explained by the fact that only one of the SNP from each of the genes (CAPN316 from the μ-calpain gene, and UASMS2 from the leptin gene) had a significant effect on any of the traits in the single SNP analyses. Thus incorporating haplotype information would not improve the performance of marker-assisted selection for this population.

## Conclusion

The results presented here confirm some of the previously documented associations, for example, the association between *CAPN316 *genotype and tenderness, the most important quality trait for consumers. Furthermore, novel associations have been identified which, following validation in other populations, could be incorporated into breeding programmes to improve meat quality. Finally, whilst some previously noted associations were not replicated in the current study, it is important to note that validation is dependent on the specific nature of the population screened and that genetic background may influence the size of the effect of a polymorphism. Validation failure may be due to a lack of true associations between the trait and marker but could also be caused by differences in SNP frequencies, different marker-causative mutation linkage phases, genotype-by-environment interactions or epistasis as well as sample size effects and the way the trait is measured. Nevertheless, for those associations confirmed here, the additional validation instils confidence in using these markers in selection programmes for improved meat quality.

## Competing interests

The authors declare that they have no competing interests.

## Authors' contributions

JLG carried out data analysis and drafted the manuscript. SCB participated in the design of the study and statistical analysis and helped draft the manuscript. CMcC participated in the design of the statistical analysis and helped to draft the manuscript. JLW conceived of the study, and participated in its design and coordination and helped to draft the manuscript. PW conceived of the study, and participated in its design, coordination and statistical analysis and helped to draft the manuscript. All authors read and approved the final manuscript.
